# Deep Learning-Based Automatic Segmentation of Ischemic Stroke Lesions in CT Perfusion Imaging

**DOI:** 10.3390/biomimetics11050334

**Published:** 2026-05-11

**Authors:** Lida Zare Lahijan, Saeed Meshgini, Reza Afrouzian

**Affiliations:** Department of Biomedical Engineering, University of Tabriz, Tabriz 51666-16471, Iran

**Keywords:** CT images, CNN, deep learning networks, ischemic stroke lesion, biomimetic systems

## Abstract

Ischemic stroke, a major cause of global disability, is characterized by the blockage of an artery leading to reduced cerebral blood flow and subsequent brain injury. Automatic segmentation of ischemic stroke lesions in Computed Tomography Perfusion (CTP) maps is critical for accurate diagnosis, treatment planning, and outcome assessment. However, the accuracy of traditional methods remains limited, with Dice Similarity Coefficient (DSC) values around 68%. To address this challenge, we propose a deep learning-based model inspired by biological systems and brain mechanisms, which emulates natural information processing to enhance ischemic stroke lesion segmentation. The proposed network architecture consists of five graph convolutional layers that automatically extract and classify features from CTP images. We evaluated the model using the ISLES 2018 database, achieving a DSC of 75.41% and a Jaccard Index of 74.52%, representing significant improvements over previous methods. Notably, the proposed approach performs robustly in noisy environments, maintaining accuracy above 60% even at SNR = −4. These results demonstrate the potential of biomimetic-inspired networks for automatic ischemic stroke segmentation.

## 1. Introduction

Stroke impacts approximately 15 million people globally each year, representing a major cause of death and long-term disability [1]. Among stroke survivors, nearly 50% develop chronic impairments [2]. Ischemic strokes, which account for roughly 90% of all cases, occur when an artery is blocked, leading to reduced cerebral blood flow, ischemia, and infarction [3]. On the other hand, type II strokes result from the rupture or tear of a weak blood vessel [4]. According to a report by the U.S. The Food and Drug Administration reports that ischemic strokes account for 87% of cases in the United States, with hemorrhagic strokes making up the remaining 13%. Various factors, including age, gender, race, and ethnicity, significantly influence the main causes of stroke.

In biological systems, the brain naturally processes information through neural networks and responds appropriately to inputs. Drawing inspiration from this natural process can help us develop systems that perform better when facing noisy and complex data. Stroke diagnosis involves a comprehensive assessment that includes thorough medical history, physical and neurological exams, along with brain imaging techniques like Computed Tomography (CT) scans or Magnetic Resonance Imaging (MRI). These diagnostic tools are crucial for ruling out alternative conditions, such as brain tumors or drug-induced disorders [5,6].

Traditional ischemic stroke lesion assessment has historically relied on both expert manual interpretation and conventional computer-aided image analysis methods. In manual practice, neuroradiologists visually inspect CT or MRI scans to identify infarcted tissue and estimate lesion extent. In addition, conventional computational approaches have included threshold-based segmentation, region-growing methods, clustering techniques, atlas- or model-based methods, and handcrafted-feature-based machine learning algorithms [7,8]. These methods usually involve several key steps, namely image preprocessing for noise reduction and intensity normalization, candidate lesion region detection, feature extraction, lesion classification or segmentation, and post-processing to refine lesion boundaries. Their performance is commonly assessed using evaluation metrics such as Dice Similarity Coefficient (DSC), Jaccard Index, sensitivity, specificity, precision, and accuracy. However, because ischemic stroke lesions often vary substantially in size, location, shape, and image contrast, traditional approaches may remain sensitive to noise, low contrast, and inter-observer variability [9,10].

Although MRI-based techniques, particularly Diffusion-Weighted Imaging (DWI), Apparent Diffusion Coefficient (ADC), Fluid-Attenuated Inversion Recovery (FLAIR), and Perfusion-Weighted Imaging (PWI) [11,12], are widely used for ischemic stroke assessment and several deep-learning-based methods have been reported for these modalities, we focused on CTP in this study because CT-based imaging remains highly relevant in acute stroke workflows due to its speed, wider availability, and fewer contraindications. In addition, the ISLES 2018 benchmark is specifically structured around lesion segmentation from acute CTP data, with DWI-based lesion annotations serving as the reference standard.

Recent studies have focused on automating ischemic stroke diagnosis. For instance, Sultanpour et al. [13] employed four 2D U-Net networks to segment ischemic lesions automatically, incorporating outlier removal and data augmentation (e.g., rotation, scaling, noise addition). Liu et al. [14] utilized CTP images and Generative Adversarial Networks (GAN) to generate Diffusion Weighted Imaging (DWI) for ischemic stroke lesion segmentation. Doles et al. [15] modified U-Net networks for stroke lesion classification, employing techniques like separate image modality processing and dense connections, improving performance across varied image sizes. Wong et al. [16] introduced an advanced framework utilizing CNNs trained on CTP-based DWI to achieve high-quality lesion segmentation, earning first place in the ISLES 2018 challenge. Albert et al. [17] used an enhanced U-Net to automatically segment ischemic strokes, achieving a DSC of 49% based on the ISLES dataset. Despite their successes, limitations in lesion detection and performance were noted. Ghanmat et al. [18] introduced a new model using deep learning to segment ischemic lesions, improving DSC by 2.5% compared to previous studies. Raju et al. [19] employed U-Net networks, achieving a DSC of approximately 42%, and Sultanpour et al. [20] used a modified U-Net architecture, incorporating multi-scale CNNs and shortcut connections, which resulted in a DSC of 68%. Tan et al. [21] used advanced machine learning models for analyzing CTP data to automatically detect ischemic strokes. CTP data was utilized as the primary source for identifying damaged brain areas. This approach provided advantages such as high accuracy in detection and improved performance in complex and noisy conditions, but it also had drawbacks, including the need for high-quality data and high computational power. Ultimately, the study showed that using CTP data could enhance stroke detection accuracy, but it requires precise preprocessing and suitable computational resources for optimal performance. The study by Alirr et al. [22] in 2025 presented a deep learning-based method using Attention ResUnet for segmenting ischemic stroke lesions from CTP images. The model utilized Edge Enhancing Diffusion (EED) preprocessing to improve the accuracy of detecting damaged areas. Data from the ISLES 2018 challenge was used, and evaluation was conducted with fivefold cross-validation. The results showed that the model’s DSC was approximately 59%, indicating challenges in accurately segmenting lesions, especially in noisy and low-quality images. This study demonstrates the advantages of advanced models but still requires improvements in accuracy and performance under noisy conditions. In another recent work, Kandpal et al. [23] introduced a DenseResU-NetCTPSS architecture for the segmentation of ischemic stroke lesions using multiparametric CTP images. Their approach integrates multiple perfusion maps, including cerebral blood volume (CBV) and time-to-maximum (Tmax), to improve lesion detection. The proposed model achieved a DSC of 0.65 on the training set and 0.45 on the test set of the ISLES 2018 dataset, highlighting both the potential and the challenges of deep learning approaches in CTP-based segmentation. Furthermore, Li et al. [24] proposed an encoder–decoder deep learning architecture incorporating multi-head cross-attention mechanisms for improved feature fusion from CTP maps. Their method leverages advanced attention-based feature extraction to better capture lesion characteristics and spatial dependencies. Although promising, the study also emphasizes the need for further improvements in robustness and generalization across different datasets and imaging conditions.

Despite these advancements, the quantitative performance of many existing ischemic stroke lesion segmentation models is still limited, with reported DSC values ranging from approximately 42% to 68.1% in representative CTP-based studies. Furthermore, although these methods have shown promising results under standard conditions, quantitative evaluation under controlled noisy environments remains limited. This issue is particularly important in CTP imaging, where motion-related degradation and acquisition noise may adversely affect segmentation reliability. In the present study, the proposed framework was therefore quantitatively evaluated under different noise levels using multiple signal-to-noise ratio (SNR) settings, demonstrating its robustness under degraded imaging conditions.

To overcome these challenges, this study presents a method utilizing deep graph convolutional networks designed to enhance performance under noisy conditions, with the goal of enabling effective automatic ischemic stroke segmentation in practical applications. The contributions of this study are threefold:

(1) We construct a superpixel-based graph representation of CTP images for ischemic stroke lesion segmentation.
(2) We design a task-specific deep graph learning pipeline based on a five-layer Chebyshev GCN to classify lesion-related graph regions.
(3) We evaluate the robustness of the proposed framework under noisy conditions, which is particularly important in CTP imaging due to motion-related degradation.

The structure of the rest of this paper is outlined as follows: Section 2 explores the dataset used and the mathematical theory behind graph convolutional networks. Section 3 introduces the proposed methodology. Section 4 provides an in-depth analysis of the simulation results obtained using the proposed approach, while Section 5 summarizes and concludes the study.

## 2. Materials and Methods

This section starts by providing an overview of the dataset used for ischemic stroke maps. Subsequently, the mathematical foundation of graph convolutional networks will be explored.

### 2.1. ISLES 2018 Database

**Figure 1:**
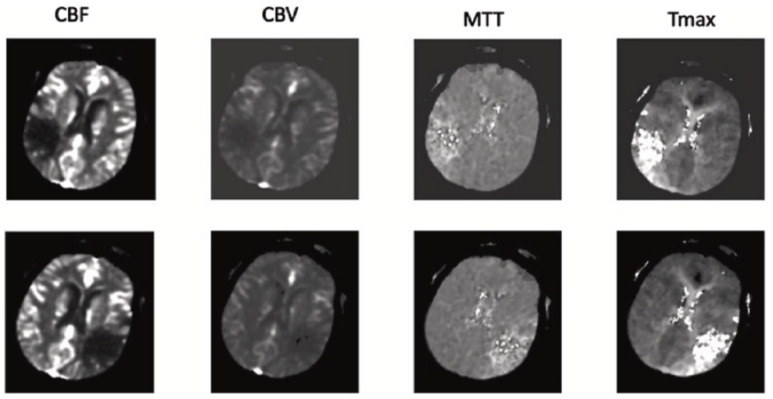
CTP image and corresponding lesions in the ISLES 2018 dataset [25].

The most complete dataset for automatic ischemic stroke segmentation has been identified as ISLES 2018. This dataset can be thought of as a derivative of the other public databases. In order to segment the core of the stroke lesion, 103 patients’ CTP scans were used to create the dataset in this database. Additionally, this collection contains 3D CTP maps with MTT, CBF, CBV, and T-max, as well as 4D CTP scans. An illustration of one of these maps is shown in Figure 1. Every patient in this database had DW-MRI for radiologist validation within three hours of basic CTP imaging. At a distance of 5 mm and a resolution of 256 × 256, the images are taken in sections with a variable number of axial slices, varying from 2 to 22 pieces depending on the patient [25].

The ISLES 2018 dataset is widely recognized as a benchmark dataset for ischemic stroke lesion segmentation and prediction tasks. It includes multiparametric CTP imaging along with lesion annotations derived from DWI, allowing supervised training and quantitative evaluation. Numerous deep learning approaches have been developed and validated on this dataset, including multi-scale U-Net variants, GAN-based image synthesis frameworks, and attention-based architectures for multimodal feature fusion. These studies highlight the importance of ISLES 2018 as a standardized platform for evaluating segmentation performance and comparing different algorithmic designs [25].

### 2.2. Standard Chebyshev Graph Convolution Background

The spectral graph convolution formulation used in this work follows the standard Chebyshev-based GCN framework reported in prior studies. The novelty of the present work lies not in redefining the graph convolution itself, but in developing a stroke-specific graph construction and segmentation pipeline for CTP lesion analysis.

The foundational concept of Graph Convolutional Networks (GCN) was introduced by Michael Deferard and his team in 2016 [26]. This work marked the first application of signal processing and graph spectral theory to graphs, which enabled the derivation of convolutional functions and the use of convolutional networks within the framework of graph theory. In graph theory, two key matrices, the adjacency matrix and the degree matrix, play a crucial role. The adjacency matrix defines the connections between vertices in the graph, while the degree matrix can be derived from it. The diagonal elements of the degree matrix represent the number of edges connecting to each corresponding vertex. The degree matrix can be expressed as **D**, and the adjacency matrix as **A**. The *i*-th diagonal element of the degree matrix is defined as follows.

(1) $$D_{\mathrm{ii}}=\sum_{j}A_{ij}$$

Since D is a diagonal matrix, its off-diagonal elements are zero, i.e., **D**_ij_ = 0 for *i* ≠ *j*.

The Laplacian matrix is defined by the following relationship:

(2) L=D−A

This equation defines that the Laplacian matrix is obtained by subtracting the adjacency matrix from the degree matrix, and it is essential for calculating graph basis functions. These functions can be derived via Singular Value Decomposition (SVD) applied to the Laplacian matrix. Additionally, the Laplacian matrix can be expressed using the eigenvector matrix and singular value matrix, as represented in the following Equation (3):

(3) $$L=U\Lambda U^{T}$$

where **U** denotes the matrix of eigenvectors, and **Λ** is the matrix of eigenvalues.

Furthermore, the Fourier transform of a graph signal can be expressed in terms of the eigenvectors, with Fourier bases defined by the diagonal eigenvalues, **λ**, as shown in the relation below:

(4) $$F(\lambda )=U^{T}f$$

To aid in understanding, the Fourier transform and its inverse for a signal like **f** can be expressed in Equations (5) and (6), respectively:

(5) $$F=U\Lambda U^{T}f$$

(6) $$f=U\Lambda^{-1}U^{T}F$$

Equation (7) represents the Fourier transform of the graph, while Equation (8) illustrates the feature vector of a signal in the context of Fourier bases and the graph’s Fourier transform. The graph convolution operator can also be computed by performing the convolution of two signals within the graph domain using the Fourier transforms of each signal. The convolution of two signals, ***z*** and ***y***, with the operator is defined as follows:

(7) $$(Z*Y)(\lambda )=U^{T}z.U^{T}y$$

In this equation, the filter function is used to define the graph convolution operator when integrated with neural networks. Thus, ***z*** is the filtered version of the signal ***y***:

(8) z=W*y

Finally, by decomposing the Laplacian matrix into its singular values and eigenvectors, graph convolution is formally defined as [26,27,28]:

(9) $$z=\sum_{i}\Lambda_{i}U_{i}^{T}y$$

In this study, the above spectral graph convolution formulation is used as the mathematical foundation of the proposed model. Specifically, each input CTP image is represented as a graph, where nodes correspond to superpixel regions obtained via the SLIC algorithm, and edges encode spatial relationships between neighboring regions. The feature vector associated with each node represents the average intensity of the corresponding region. Based on this graph representation, the Chebyshev polynomial approximation of graph convolution is applied to propagate and aggregate information across neighboring nodes. This allows the model to capture both local and contextual dependencies between different regions of the image, which is essential for accurate segmentation of ischemic stroke lesions.

### 2.3. General Model of Simple Linear Iterative Clustering (SLIC) Algorithm

The SLIC algorithm is employed to divide the input image *I* into several distinct regions, forming an adjacency graph ***G***. The number of regions, denoted as *k*, is determined randomly, while the spatial resolution is defined by the product *P* × *Q*, where *P* and *Q* represent the image’s dimensions. The image features are normalized to the range [0, 1] by dividing each channel by the bit depth *B*, which corresponds to the number of bits used for each color channel [20,29].

In the resulting neighborhood grid, superpixels *R_i_* are represented as vertices *V_i_*, each associated with a one-dimensional feature vector ***F_i_*** that reflects the characteristics of the corresponding region. The average pixel intensity for each superpixel *R_i_* is computed by taking the mean intensity of all pixels within the region. Adjacent superpixels are linked by weighted edges, where the weights are determined using a Gaussian weighting function. The weight ***W**_ij_* between two adjacent regions is calculated as

(10) $$W_{ij}=\exp(-\frac{d^{2}}{2\sigma^{2}})$$

where *d* is the spatial distance between regions, and *σ* is a parameter that controls the sensitivity of the weight.

The Gaussian weighting function is adopted to model the spatial relationship between adjacent regions in a smooth and continuous manner. This formulation assigns higher weights to spatially closer regions while gradually reducing the influence of more distant regions, which is consistent with the local continuity assumption in medical images. Moreover, the Gaussian kernel helps reduce the impact of noise and intensity fluctuations by limiting the contribution of less relevant or distant regions, thereby improving the robustness of the graph representation.

The adjacency matrix ***A***, with size *N* × *N*, captures the relationships between regions and is given by:

(11) $$A=[a_{ij}]\in {\mathbb{R}}^{N\times N}$$

Additionally, there is a region feature matrix **x** ∈ $X\in {\mathbb{R}}^{N\times 1}$, which holds the features of the *N* vertices in the graph, with each vertex representing the characteristics of the respective region [30].

## 3. The Suggested Model

**Figure 2:**
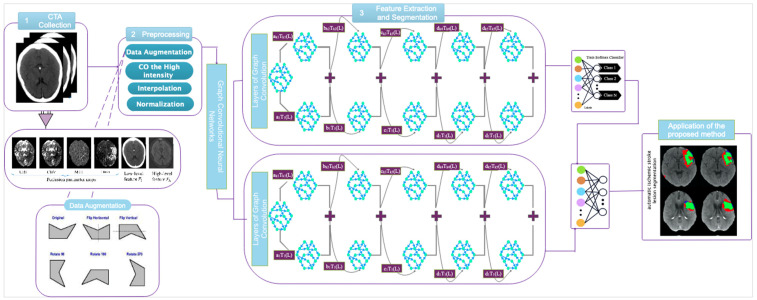
Schematic block diagram of the proposed deep learning-based framework for automatic segmentation of ischemic stroke lesions in CT perfusion imaging.

This section presents the methodology proposed in this paper. The main structure of the suggested model is illustrated in Figure 2.

### 3.1. Pre-Processing of Data

Initially, each image undergoes pre-processing by taking into account a Hounsfield coefficient. Pixels with intensities greater than 150 and less than zero are eliminated and replaced with zero. Next, the matching portion of 256 × 256 is read in order to determine the size of the images. After that, to improve generalization and reduce overfitting, data augmentation was applied to the training images. The augmentation techniques included rotation within the range of −20° to +20°, as well as horizontal flipping. These transformations were applied only to the training set and not to the validation or test sets.

### 3.2. Create a Graph

**Figure 3:**
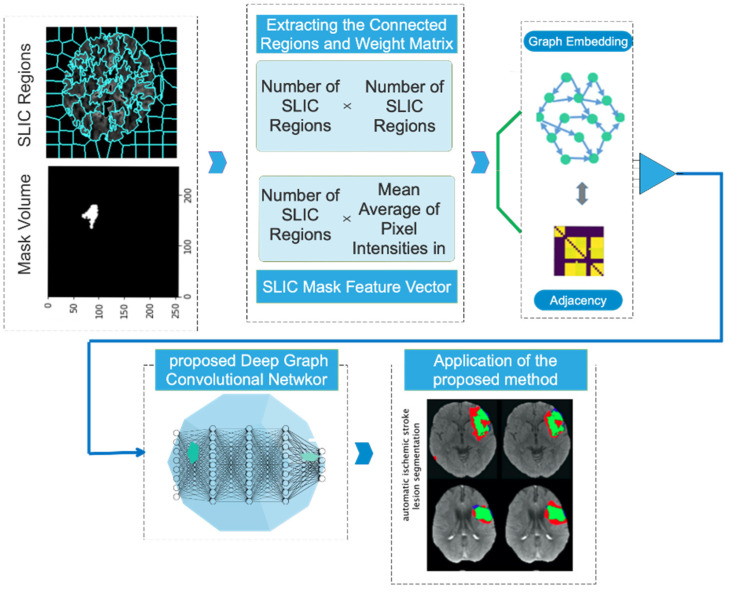
Schematic workflow illustrating graph construction and the integration of SLIC superpixel segmentation for ischemic stroke lesion analysis.

The SLIC approach is used to obtain the graph representation. Using this technique, a number of superpixels that represent different areas of the image are extracted from a cluster of images. The steps involved in making a graph with this approach are shown in Figure 3. The relationship between each node in this network and the superpixel areas that were recovered using the SLIC technique is shown in Figure 3. Additionally, the feature vector for each region and network node is calculated based on the average pixel intensity within that region. Furthermore, the edges of the graph are assessed according to how close and far they are from each region while creating a graph adjacency matrix. When creating a graph, adjacent regions are connected, but non-neighboring regions are not.

### 3.3. Proposed Deep Network Design

**Figure 4:**
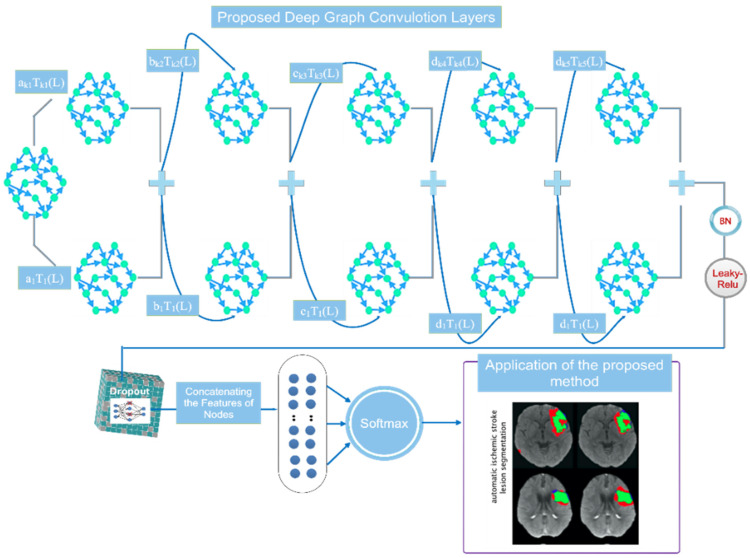
Detailed architecture of the proposed network, illustrating its main layers, feature extraction modules, and interconnections.

The proposed deep network architecture is illustrated in Figure 4. The overall design of the model is built upon a superpixel-based graph representation of CTP images, where each node corresponds to a segmented image region and each node feature encodes the local characteristics of that region. Based on this graph formulation, a deep Chebyshev graph convolutional network is employed to perform automatic lesion region classification. More specifically, the network consists of five stacked graph convolutional blocks. In each block, graph convolution is performed using the Chebyshev spectral formulation in order to aggregate information from neighboring nodes while preserving the underlying graph topology. This choice enables the model to efficiently capture both local structural relationships and regional contextual information across the superpixel graph. After each graph convolution operation, batch normalization is applied to stabilize feature distributions during training and improve convergence. A Leaky ReLU activation function is then used to introduce nonlinearity and prevent inactive gradient behavior that may arise with conventional ReLU units. An additional batch normalization layer is further employed to improve training robustness and reduce internal covariate shift. The same graph convolutional block is repeated five times to progressively learn higher-level and more discriminative representations from the graph-structured CTP data. The use of multiple stacked graph convolutional layers allows the network to incorporate broader contextual information from adjacent and higher-order neighboring regions, which is particularly important in ischemic stroke lesion segmentation, where lesion boundaries may be diffuse and spatially heterogeneous.

Following the graph feature extraction stage, a dropout layer is introduced to reduce the risk of overfitting, especially considering the relatively limited size of the ISLES 2018 dataset. This regularization step improves the generalization capability of the proposed model. The output features are then forwarded to a fully connected classification stage, where the Softmax activation function is used to generate the final class probabilities for lesion and non-lesion regions. Overall, the proposed architecture is not intended to redefine the mathematical basis of graph convolution, but rather to provide a task-specific deep graph learning framework tailored to ischemic stroke lesion segmentation in CTP images. By combining superpixel-based graph construction with a multi-layer Chebyshev graph convolutional design, the model is able to exploit both regional image characteristics and graph-topological relationships for more robust lesion identification.

**Figure 5:**
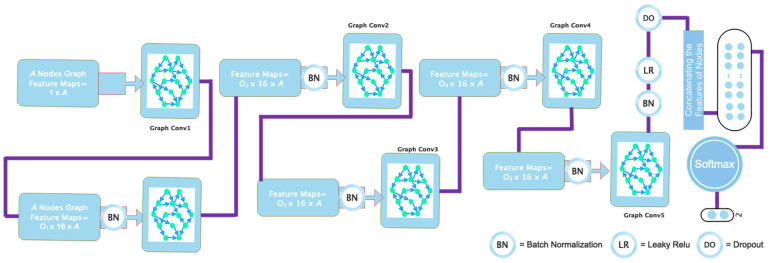
Schematic representation of the proposed deep network architecture, including the detailed specifications of its layers.

Figure 5 illustrates the differences between the various layers. Each node in the constructed network corresponds to a single instance, with its feature vector representing the average pixel intensity in each region. The graph convolution layer in the proposed architecture has an input dimension of 16 pixels for all network nodes. The first layer’s output dimension is also 16, resulting in a graph with vertices labeled A and 16 samples per node. As shown in Figure 5, the graphs generated in the previous step are used as input for the feature encoder stage, indicated by node A. The second graph convolution layer then produces an A-node graph with sixteen samples per vertex. This process is repeated iteratively until the fifth graph is generated, yielding an A-node graph with a dimension of two.

After this, the output from the feature encoding stage passes through a dropout layer. After being flattened with two samples per node, the resultant A-node graph is converted into a vector of A elements and fed into a Softmax layer. Additionally, Figure 5 shows that the coefficients O1, O2, O3, and O4 for the five designated graph convolutional layers define the specific order of each layer in the Chebyshev polynomial expansion. Table 1 provides a comprehensive summary of the network’s specifications and the details of each layer.

### 3.4. Training and Test Series

In this study, the proposed network was trained and evaluated using the 10-fold cross-validation method through trial and error. This approach prevents overfitting during training and allows all samples to take part in the evaluation. In this study, 10-fold cross-validation was performed at the patient level, such that all slices belonging to a given patient were assigned to the same fold. This strategy was adopted to prevent data leakage between training and testing sets.

The ideal criteria used for the suggested architecture’s design are displayed in Table 2. This table makes it evident which optimization techniques, how many layers there are, etc., have all been tried, and the optimal solution has been chosen for the suggested design.

## 4. Results

The outcomes of the proposed method are presented in this section. All simulations were performed in the Google Colaboratory environment using a Tesla K90 GPU with 25 GB of RAM. The implementation included author-developed components, such as graph construction from CTP images and the proposed network architecture, together with established approaches for Chebyshev-based graph convolution and standard training procedures.

**Figure 6:**
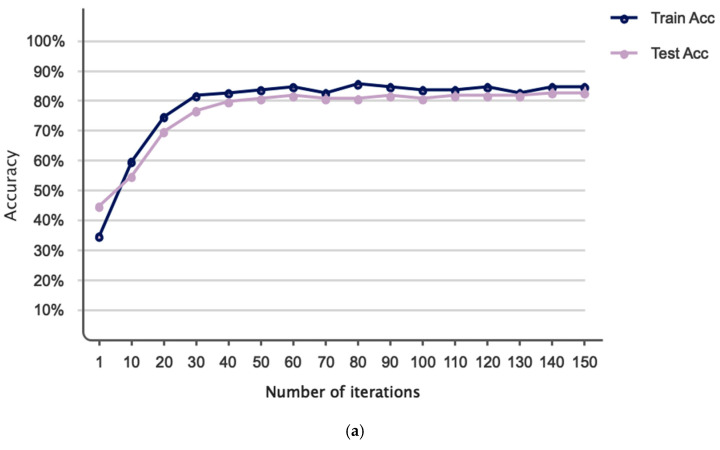

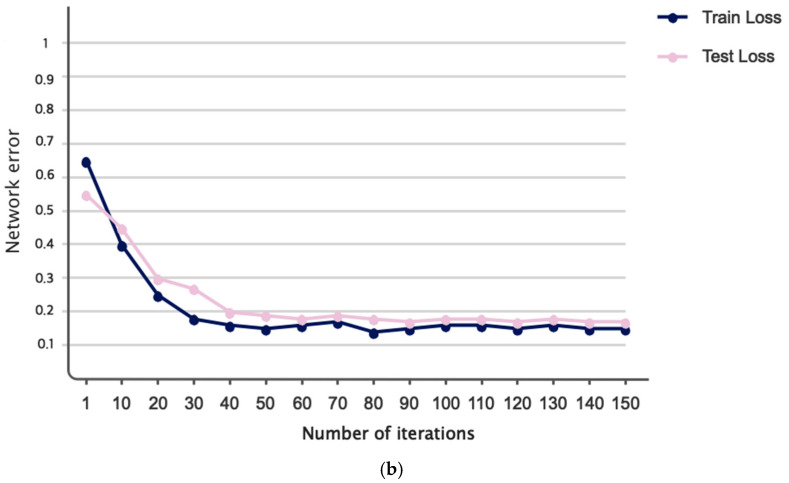
Model performance across different iterations: (**a**) accuracy and (**b**) error metrics.

**Figure 7:**
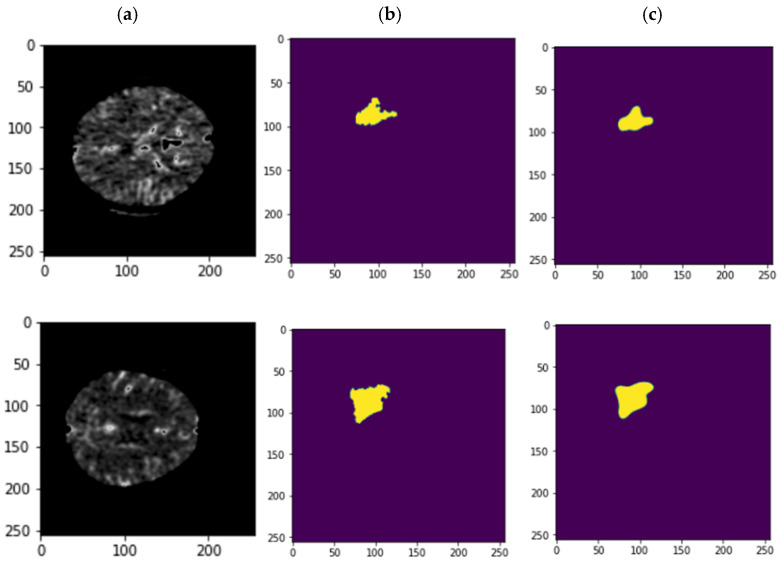
Comparison CT image (**a**), corresponding mask (**b**), and performance of the proposed model in diagnosing ischemic stroke (**c**).

**Figure 8:**
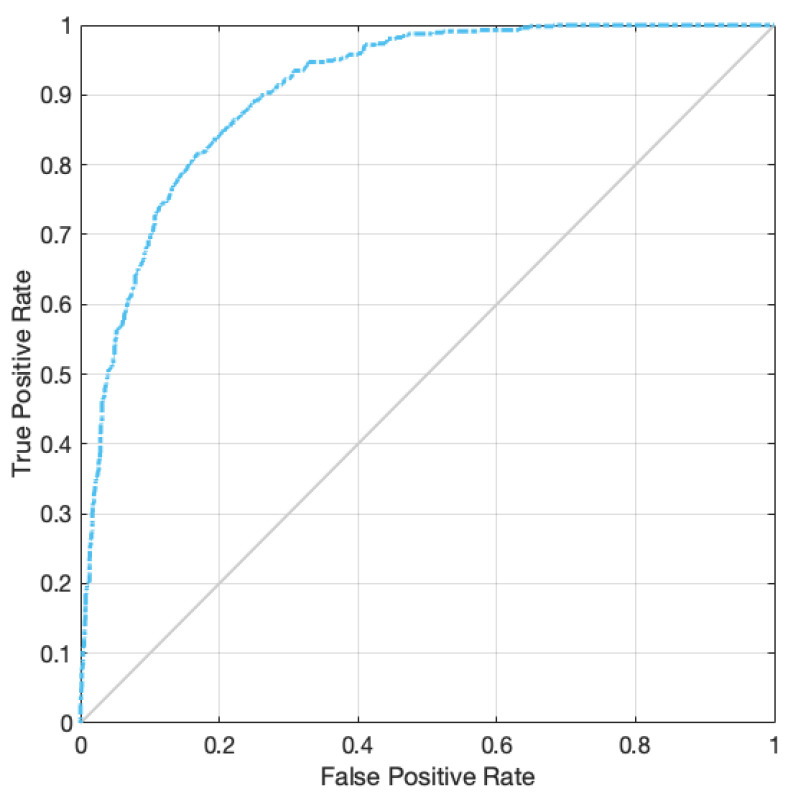
ROC curve analysis for automatic segmentation of stroke lesions.

**Figure 9:**
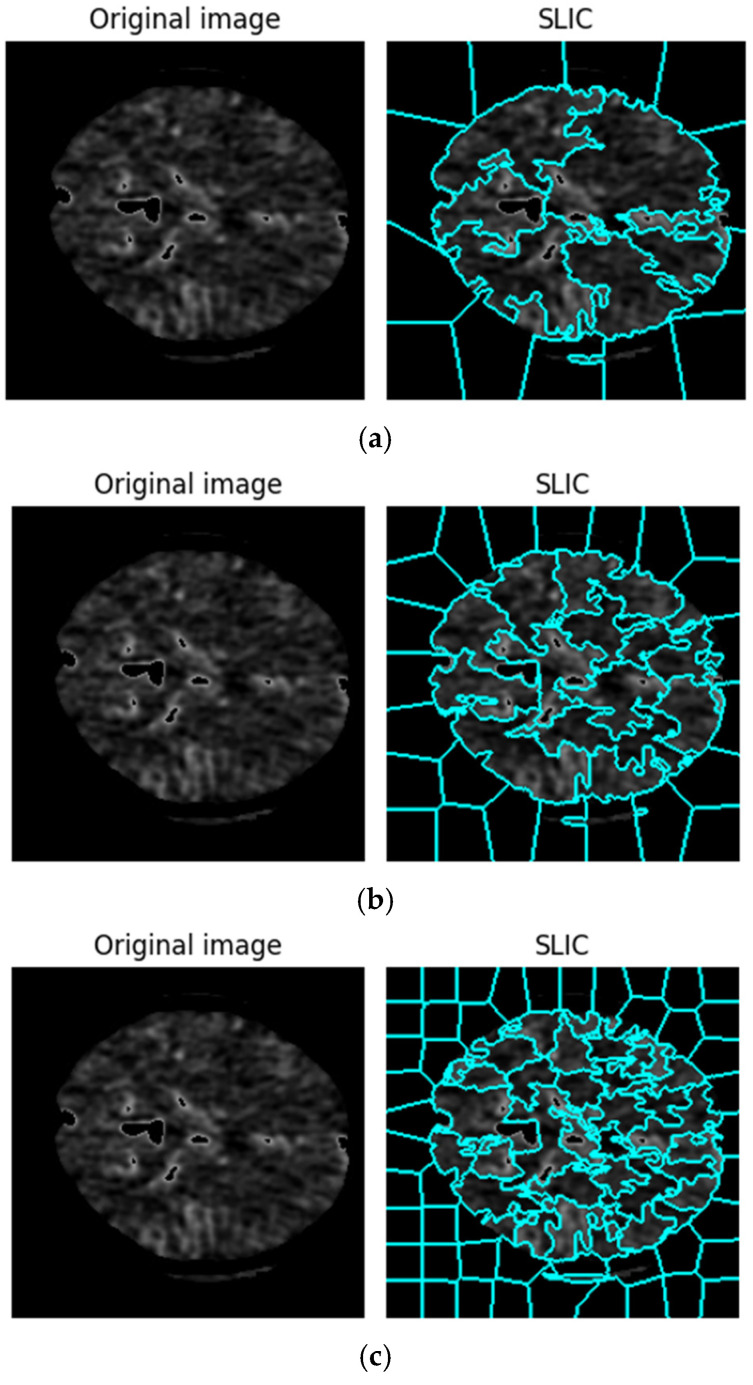

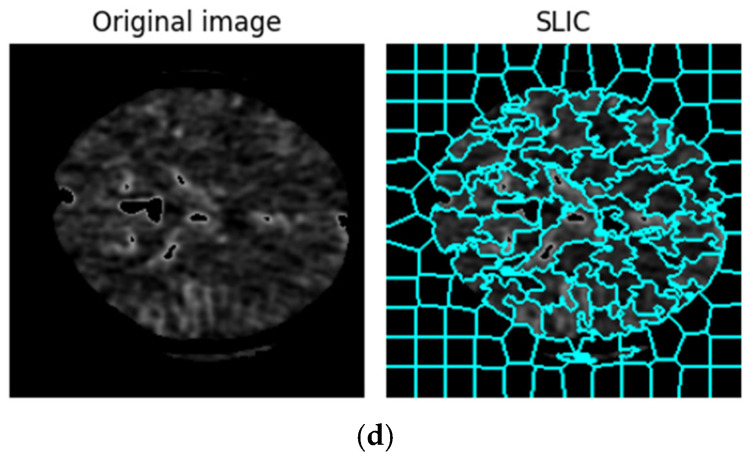
Regions selected by SLIC algorithm: (**a**) 25, (**b**) 50, (**c**) 100, (**d**) 150 zones.

The accuracy and error of the suggested technique for automatically segmenting an ischemic stroke lesion are displayed in Figure 6. The chart indicates that 150 repeats are taken into account for training and evaluating the network; as the number of these repetitions increases, so does accuracy and error. According to this result, the network’s accuracy has now reached 83% for the test set. Figure 7 depicts the primary instances of CT images, binary masks, and predicted images generated by the trained network for automatic ischemic stroke segmentation, from left to right. Since ischemic stroke lesion segmentation is a highly imbalanced problem, DSC and Jaccard Index were considered the primary performance metrics in this study, while accuracy was reported only as a supplementary measure. As shown in Table 3, the proposed model achieved a DSC of 75.41%, a Jaccard Index of 74.52%, a sensitivity of 78%, and a precision of 80.89%. These results indicate that the proposed graph-based framework provides competitive lesion segmentation performance on the ISLES 2018 dataset. Also, to assess the statistical significance of these performance metrics, we performed paired t-tests and Wilcoxon signed-rank tests. The *p*-values reported for DSC, Accuracy, Sensitivity, and Precision indicate that the improvements observed in these metrics are statistically significant, with *p*-values less than 0.05 for all metrics. This suggests that the proposed method provides robust and reliable performance in ischemic stroke lesion segmentation. Notably, these results surpass the performance of several prior U-Net-based models, confirming the effectiveness of the proposed method in automatically segmenting ischemic stroke lesions from CT perfusion images. Figure 8 depicts the results of a Receiver Operating Characteristic (ROC) performed on the proposed network. The segmentation graph falls within the range of 0.6 to 0.9, demonstrating that the proposed network achieves optimal performance, according to the evaluation criteria. Figure 9 depicts CT image grouping with varying numbers of zones. For visual comparison, clustering based on 25, 50, 100, and 150 zones is displayed individually. To obtain the matching graph, 100 zones were chosen to strike a balance between the areas of the SLIC approach and segmentation accuracy. Table 4 shows the details of the evaluation parameters for these three zones. As shown in Table 4, increasing the number of zones beyond 100 does not improve performance and may even degrade it due to fragmentation of meaningful regions. Therefore, 100 zones were selected as the optimal configuration, providing a balance between spatial detail and segmentation accuracy.

**Figure 10:**
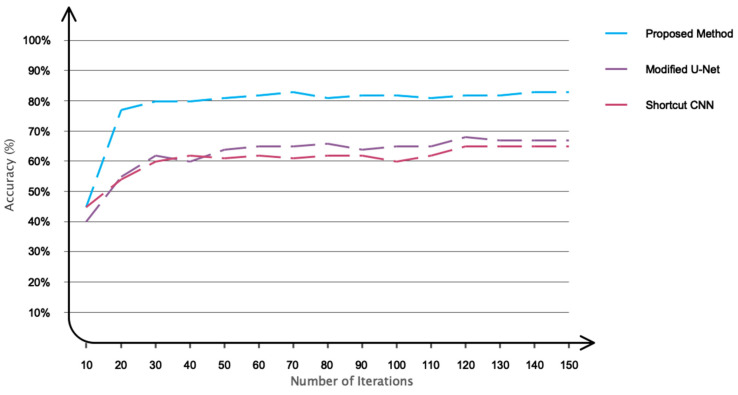
Comparing the performance of the present study with other common networks.

To assess the performance of the proposed model for ischemic stroke segmentation, a comparison was made with the Modified U-Net and Shortcut CNNs [25,26], both of which have been extensively used in recent studies on ischemic stroke segmentation. The accuracy performance results are presented in Figure 10. As illustrated, the proposed network outperforms the others in terms of accuracy. Additionally, it exhibits a significantly lower rate of fluctuation when compared to the two analyzed networks, which can be attributed to the customized design of the proposed architecture.

Furthermore, the effectiveness of the proposed method was compared with results from recent studies, with the collected data summarized in Table 5. The findings reveal that the proposed method for automatic ischemic stroke segmentation achieved a DSC rate of 75.41%. In contrast, the same metric in studies [19,20] were 68% and 42%, respectively. The proposed method achieved a DSC of 75.41% and a Jaccard Index of 74.52%, indicating competitive segmentation performance on the ISLES 2018 dataset in the adopted 2D slice-wise evaluation setting. The table also highlights the strengths of our approach, including its superior performance in maintaining segmentation accuracy despite the presence of noise, making it a promising solution for real-world clinical applications.

The position, size, and severity of the lesion can be challenging for the attending physician to diagnose if the patient moves slightly, as CTP images are highly sensitive to patient movement. Regrettably, previous research has not assessed their proposed models in noisy conditions, likely due to the decline in algorithmic performance in such scenarios. It is well-known that testing and evaluating a proposed model under various uncertainties, including noise, is essential before it can be applied in clinical practice. Therefore, in this study, Gaussian white noise was added to the images at various SNR levels, ranging from −4 dB to 5 dB, to simulate the effect of patient motion and image degradation. The lower SNR levels (closer to −4 dB) represent scenarios with high noise (mimicking significant patient motion), while higher SNR levels (closer to 5 dB) simulate lower noise and more stable imaging conditions.

**Figure 11:**
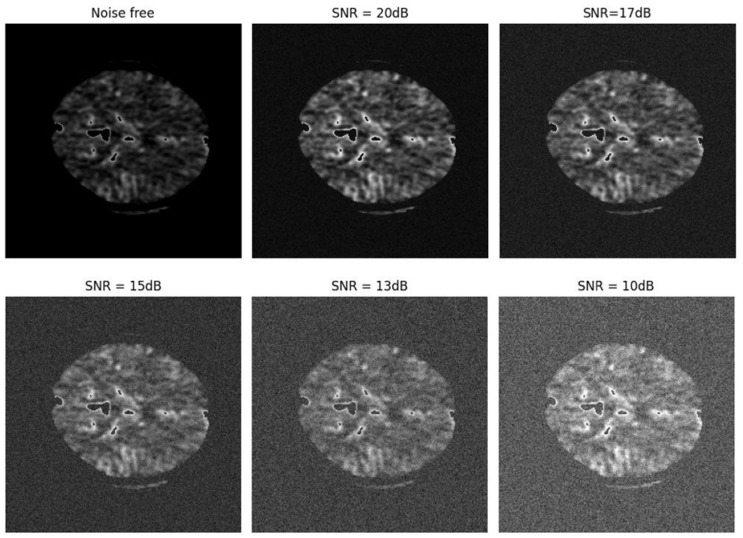
Noise levels applied to the images at varying Signal-to-Noise Ratios (SNRs).

**Figure 12:**
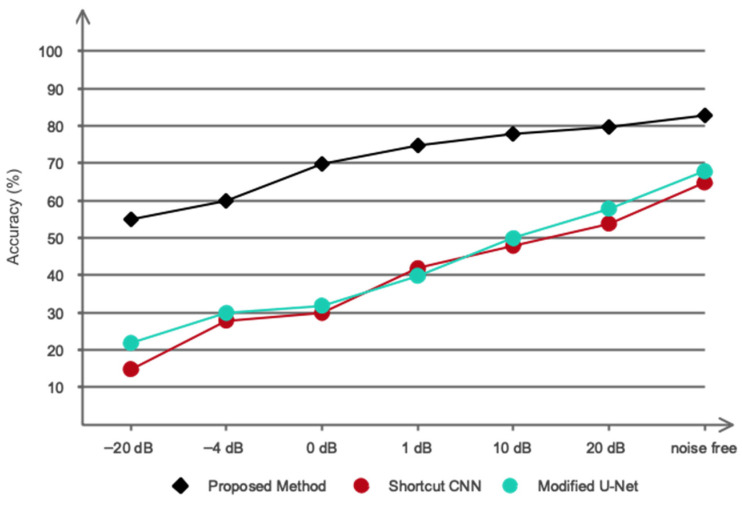
The proposed network’s performance was benchmarked against other existing networks to evaluate its relative effectiveness.

Figure 11 shows the noise applied to the images at different SNR levels, and Figure 12 presents the segmentation results obtained using the proposed model as well as the Shortcut CNN and Modified U-Net. These results clearly demonstrate that the proposed network exhibits strong resilience across a wide range of SNR values, effectively maintaining segmentation accuracy even in highly noisy conditions.

Recent advances in data-driven biomedical research and applied computational modeling have increasingly demonstrated the value of artificial intelligence and quantitative analysis across diverse clinical and scientific domains, including epidemiological estimation using machine learning [31], disease comorbidity graph analysis [32], electronic health record-based disease clustering [33], spatial transcriptomics analysis [34], therapeutic mechanism and translational medicine studies [35], biomaterial surface engineering and biocompatibility assessment [36,37], hematologic oncology case analysis [38], nanomedicine-based anticancer strategies [39], cardiac SPECT imaging optimization [40], health-system indicator modeling [41], algorithmic pathfinding enhancement [42], digital health service evaluation [43], surface roughness prediction [44], resilient network optimization [45], high-speed spindle bearing performance improvement [46], indigenous knowledge transmission frameworks [47], vegetation and climate modeling [48], ecological impact assessment [49], passive environmental design [50], photocatalytic air purification materials [51], thermal photonics [52], emergency radiology evidence synthesis [53], stroke-related hemorrhagic risk evaluation after mechanical thrombectomy [54], neuropsychiatric drug development [55], rehabilitation design strategies [56], extended reality in healthcare architecture [57], mammographic tumor detection using deep learning [58], and autism diagnosis through functional magnetic resonance imaging and machine vision [59]. Within this broader interdisciplinary context, the present study, Deep Learning-Based Automatic Segmentation of Ischemic Stroke Lesions in CT Perfusion Imaging, further contributes to the growing body of AI-enabled medical imaging research by focusing on automatic lesion segmentation in CT perfusion data, with the aim of supporting faster, more objective, and more reproducible assessment of ischemic stroke lesions.

This study, like other studies, has benefits and drawbacks. One of the benefits of this research is attaining the best segmentation performance. Furthermore, giving a trustworthy method based on graph convolutional networks may be an ideal platform for ischemic stroke classification. While the proposed model shows strong performance on the available dataset, its generalization to real-world clinical settings may be influenced by dataset limitations. In practice, variations in acquisition protocols, noise characteristics, and patient heterogeneity may lead to some reduction in segmentation performance, including lower DSC values. Nevertheless, the robustness analysis conducted under controlled noise conditions provides evidence that the model maintains stable behavior under degraded imaging scenarios. A further limitation of the present study is that no direct comparison was performed against volumetric architectures such as the 3D U-Net. Therefore, although the proposed 2D graph-based model showed promising segmentation performance, no claim is made regarding superiority over fully volumetric methods. Future work will investigate the extension of the framework toward 3D graph representations and direct benchmarking against strong volumetric baselines. Due to its outstanding performance, the proposed model holds potential for future use in aiding doctors and radiologists in accurately determining the dimensions and location of ischemic stroke lesions.

## 5. Conclusions

This study presented a deep learning-based framework for automatic segmentation of ischemic stroke lesions using CTP images. The proposed approach integrates a superpixel-based graph representation with a five-layer Chebyshev graph convolutional network to effectively capture both local and contextual information from imaging data. The experimental results on the ISLES 2018 dataset demonstrate that the proposed model achieves a DSC of 75.41% and a Jaccard Index of 74.52%, outperforming several recent methods reported in the literature. In addition, the model achieved a sensitivity of 78% and a precision of 80.89%, indicating a reliable balance between detection capability and false positive control. The results also show that the proposed framework maintains robust performance under noisy conditions, highlighting its potential applicability in real-world clinical environments where image quality may be degraded. The findings suggest that incorporating graph-based representations can significantly enhance lesion segmentation performance by modeling spatial relationships between image regions more effectively than conventional pixel-based approaches. Overall, the proposed method provides a robust and effective solution for ischemic stroke lesion segmentation and shows strong potential for supporting clinical decision-making processes.

## Figures and Tables

**Table 1:** The number of filters, stride dimensions, and architectural specifications of the customized Convolutional Neural Network (CNN) model are detailed.

Layer	Shape of Weight Tensor	Shape of Bias	Number of Parameters
**First graph convolution**	(O_1_, 16, 16)	16	256 × O_1_ + 16
**Batch normalization**	(16)	16	32
**Second graph convolution**	(O_2_, 16, 16)	16	256 × O_2_ + 16
**Batch normalization**	(16)	16	32
**Third graph convolution**	(O_3_, 16, 16)	16	256 × O_3_ + 16
**Batch normalization**	(16)	16	32
**Fourth graph convolution**	(O_4_, 16, 16)	16	256 × O_4_ + 16
**Batch normalization**	(16)	16	32
**Fifth graph convolution**	(O_5_, 16, 2)	2	32 × O_5_ + 2
**Batch normalization**	(16)	16	32
**Softmax layer**	—	2	2 × A × O_4_

**Table 2:** Selected parameters in the proposed model.

Parameter	Search Space	Optimal Value
**Optimizer**	RMSprop, Adam, SGD, Adamax, Adadelta	Adadelta
**Error Function**	MSE, Cross Entropy	Cross Entropy
**Number of graph convolution layers**	3, 5, 7, 10	5
**Size of output in first layer**	16, 32, 64, 128	16
**Size of output in second layer**	16, 32, 64, 128	16
**Size of output in third layer**	16, 32, 64, 128	16
**Size of output in fourth layer**	16, 32, 64, 128	16
**Weight of optimizer**	6 × 10^−5^, 6 × 10^−4^	6 × 10^−4^
**Dropout rate**	0, 0.2, 0.3, 0.4, 0.5	0.3
**Learning rate**	0.01, 0.001, 0.0001	0.0001

**Table 3:** Results obtained in various evaluation indicators.

Measurement Index	Performance (%)	*p*-Value (*t*-Test)	*p*-Value (Wilcoxon)
Accuracy	83.1	0.002	0.003
DSC	75.41	0.001	0.004
Jaccard Index	74.52	0.003	0.004
Sensitivity	78	0.005	0.006
Precision	80.89	0.003	0.004

**Table 4:** Results obtained based on the number of different selected zones.

Measurement Index	The Number of Selected Zones			
	25	50	100	150
Accuracy	72.27	79.74	83.1	82.85
DSC	64.94	67.91	75.41	71.25
Jacard	62.25	70.01	74.52	72.42
Precision	68.87	75.25	80.89	78.92

**Table 5:** The suggested model is compared to current studies.

Research	Method	Year	DSC (%)
**Wang et al.** [16]	Multi-scale U-Net	2020	55
**Clèrigues et al.** [17]	FCN	2019	57
**Ghnemat et al.** [18]	Mutation model + GAN	2023	43.22
**Raju et al.** [19]	U-Net with Group Convolutions	2022	42
**Soltanpour et al.** [20]	MultiRes U-Net	2021	68.1
**Tan et al.** [21]	Complex CNN	2025	69
**Alirr et al.** [22]	EED + Attention ResUnet	2025	59
**Kandpal et al.** [23]	DenseResU-NetCTPSS	2025	~45
**Li et al.** [24]	CNN-based segmentation	2026	~68
**Proposed method**	Graph Convolution Network	2026	75.41

## Data Availability

The datasets used in this study are publicly available at the following address links: ISLES Challenge 2018 Ischemic Stroke Lesion Segmentation. *ISLES Challenge*. Accessed on 5 February 2026. https://www.isles-challenge.org.
